# Small is beautiful: demystifying and simplifying standard operating procedures: a model from the ethics review and consultancy committee of the Cameroon Bioethics Initiative

**DOI:** 10.1186/s12910-016-0110-8

**Published:** 2016-05-13

**Authors:** Odile Ouwe Missi Oukem-Boyer, Nchangwi Syntia Munung, Godfrey B. Tangwa

**Affiliations:** Cameroon Bioethics Initiative (CAMBIN), Yaoundé, Cameroon; Centre de Recherche Médicale et Sanitaire (CERMES), Niamey, Niger; Department of Medicine, University of Cape Town, Cape Town, South Africa; Department of Philosophy, University of Yaoundé I, Yaounde, Cameroon

**Keywords:** Africa, Research ethics committee, Standard operating procedures, WHO’s guidelines, Gold standard

## Abstract

**Background:**

Research ethics review is a critical aspect of the research governance framework for human subjects research. This usually requires that research protocols be submitted to a research ethics committee (REC) for review and approval. This has led to very rapid developments in the domain of research ethics, as RECs proliferate all over the globe in rhyme with the explosion in human subjects research. The work of RECs has increasingly become elaborate, complex, and in many cases urgent, necessitating supporting rules and procedures of operation. Guidelines for elaborating standard operating procedures (SOPs) for the functioning of RECs have also been proposed. The SOPs of well-placed and well-resourced RECs have tended to pay much attention to details, resulting, as a consequence, in generally long, elaborate, intricate and complex SOPs; a model that can hardly be replicated by other committees, equally under ethics review pressures, but working under much more constraining conditions in resource-destitute environments.

**Methods:**

In this paper, we looked at the content and length of SOPs from African RECs and compared them to the World Health Organization (WHO)’s guidelines as the gold standard. We also looked at the SOPs from the Ethics Review and Consultancy Committee (ERCC) of the Cameroon Bioethics Initiative that we elaborated in a simplified way in 2013, and compared them to the WHO’s guidelines and to the other SOPs.

**Results:**

Sixteen SOPs from 14 African countries were collected from various sources. Their average length was of 30 pages. By comparison to the guidance of the WHO, only six of them were found acceptable with more than 70 % of the criteria from the gold standard that were fully described. Among those six, two of them were very long and detailed (65 and 102 pages), while the four remaining SOPs ranged from 16 to 24 pages. The ERCC SOPs are seven pages long but maintain all that is of essence for the rigorous, efficient and timely review of protocols.

**Conclusions:**

We are convinced that, because of their brevity, simplicity, clarity and user-friendliness, the ERCC SOPs recommend themselves as a model template to, at least, committees similarly situated and/or circumstanced as the ERCC of the Cameroon Bioethics Initiative is. In fact, brevity, clarity, simplicity and user-friendliness are recognized values. Whatever is brief and clear is better than what is not and saves time. What is simple and user-friendly is better than what is not even though the two have the same aims because it saves both time and mental energy. And if this be true in general, it is even truer of the context and its peculiar constraints that we are addressing.

## Background

The global explosion in human subjects research, following the emergence of the HIV/AIDS pandemic and other life-threatening epidemics in the mid-1980s, has witnessed a corresponding explosion in research oversight and governance in view of the risks and dangers of research on human beings. A central aspect of research oversight involves the review and approval of research protocols by research ethics committees (RECs), otherwise called institutional review boards (IRBs) in some jurisdictions, before the proposed research is carried out. The Declaration of Helsinki [1], generally acknowledged as the preeminent regulatory document for research involving human subjects, recognizes the fact that “Medical progress is based on research that ultimately must include studies involving human subjects (Article 5) [1], but insists that “The design and performance of each research study involving human subjects must be clearly described in a research protocol. …” (Article 22) and that “The research protocol must be submitted for consideration, comment, guidance and approval to the concerned research ethics committee before the study begins. …” (Article 23). Ethics review has thus become an important pillar of the oversight and governance framework and RECs have become an indispensable part and parcel of all research involving human beings. However, the task of protocol review and the work and functioning of RECs/IRBs has become quite complex and evolving in the last couple of decades. In some parts of the world, notably the industrialized Western world, protocol review and the functions of RECs has become an almost independent ‘industry’ in synergy with the ever expanding volume and varieties of human subject research around the globe and the challenges and controversies arising from or connected with it. Like with many other things Western, a lively and rapidly expanding theoretical field and specialization is growing around research ethics and protocol review. In other parts of the world, new RECs/IRBs are increasingly springing into existence to provide local oversight to local or collaborative international research [2].

All this has led to attempts at streamlining and uniformizing the procedural rules and practices connected with research ethics review in all parts of the world. Such attempts aim at articulating standards that delineate what is required as a minimum for committees to meet globally agreed upon benchmarks in at least the core elements of ethical review, operations, independence and governance. These attempts can be seen in various guidelines for elaborating standard operating procedures (SOPs) for ethics review committees. A case in point is the World Health Organization (WHO)’s “Standards and Operational Guidance for Ethics Review of Health-Related Research with Human Participants” [3]. Standard Operating Procedures are important for normal functioning of ethics committees and in some cases required for accreditation of RECs, although they are generally legally non-binding.

The general situation of human research ethics review in Africa (excepting perhaps only the Republic of South Africa and a couple of other countries) is characterized by poor regulation, inexistent or weak legislation, inexistent or obsolete infrastructure and lack of expertise; hence the urgent need for capacity building in health research ethics, particularly in review committee training towards effective and efficient protocol review [4–6]. It was our underlying intuitive idea that long and detailed, let alone intricate and complex SOPs, tend to mystify protocol review and may be a hindrance rather than a help in capacity building in research ethics review in Africa, particularly in Central and West Africa, where we have been involved in or connected with such capacity building [7].

In order to verify this idea, we then sourced for SOPs from various RECs in Africa, and analyzed their content in relation to the WHO's guidelines. We also looked at the SOPs from the Ethics Review and Consultancy Committee (ERCC) of the Cameroon Bioethics Initiative (CAMBIN) that we elaborated in a simplified way in 2013, following the WHO’s guidelines as a standard and then compared these to the other SOPs.

## Methods

### Collection of standard operating procedures from African research ethics committees

The Council on Health Research for Development (COHRED)’s Health Research Web (*HRWeb*) platform [4, 8, 9] was used to undertake a systematic search of mapped RECs in Africa and to download SOPs when they were made available. In addition, we collected SOPs from REC websites, and received hard or soft copies from personal contacts.

### Qualitative analysis of collected standard operating procedures

The SOPs were then numbered to preserve anonymity for further analyses and presentation of results. The numbers of pages, annexures and total number of pages were registered for each SOP. Then, the content of each SOP was qualitatively analyzed for the presence or absence of the 23 criteria that are recommended in the WHO's guidelines [3], which, in this study, we considered as the gold standard. To do this, a color code was used to report the complete (green), incomplete (orange) or absence (red) of these criteria in each of the SOPs. Two of the authors independently did this content analysis and compared the results. Discrepancies were discussed and then solved by evidence-based consensus. For example, if one investigator marked that SOP 1 had criterion 23 and this was different from that the second investigator, the first investigator would indicate the page on the SOP that she felt justified her choice and the reason. For each SOP, the percentages of the complete, incomplete or absence of description of these criteria were calculated. All 23 criteria were given the same weight. In our grading system, the scale goes from fail (below 50 %) through pass (50–59 %) through fairly good (60–69 %) through good (70–79 %) to very good (80–100 %). We then considered that an acceptable SOP must have at least 70 % of criteria that are described completely. Based on the above analysis, we analyzed the 23 criteria in order to see which were those that were always, often, infrequently or never described in the collected SOPs.

### Focus on the standard operating procedures of the ethics review and consultancy committee of the Cameroon bioethics initiative

In 2011, the Cameroon Bioethics Initiative (CAMBIN) started developing SOPs for its Ethics Review and Consultancy Committee (ERCC), with the principal objective of maintaining standards in its activities. These SOPs were completed in 2013. They outline procedures, using the WHO’s standards and guidance for ethics review committees as a template, for governance, the review process and monitoring of approved proposals. The SOPs of the ERCC are a 7- page document (excluding annexures), which has been developed with the mindset of making the procedures concise and clear, so that investigators and members of the ERCC could easily comprehend and follow them.

## Results

### Collection of standard operating procedures from African research ethics committees

**Fig. 1:**
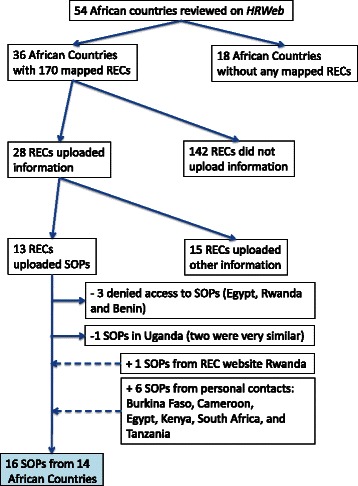
Flowchart summarizing the collection of Standard Operating Procedures from African Research Ethics Committees. HRWeb: Health Research Web, SOPs: Standard Operating Procedures, RECs: Research Ethics Committees

Between May 2013 and February 2014, we systematically visited the *HRWeb* Regulation and Ethics Review of Research pages of each African country [4]. Of the 54 African countries listed, 36 countries were mapped, totaling 170 RECs, whereas 18 countries had no REC mapped (Fig. 1). Among the 170 RECs, 28 RECs from 19 countries uploaded additional information, including but not limited to internal regulations, mission statement, flyers, submission checklist, researcher’s guidelines, ethics clearance proposal form, and SOPs. Among these 28 RECs, the latest upload of information or file was between 2 months and 3 years back (average 2,04 years). Thirteen RECs from 11 different countries posted SOPs on the *HRWeb*; however, access to these SOPs was denied for 3 of these RECs, namely the Comité d’Ethique de la Recherche de l’Institut des Sciences Biomédicales Appliquées in Benin, the IRB of Theodor Bilharz Research Institute in Egypt and the Rwanda National Ethics Committee. For Uganda, two listed RECs were very similar and so only one document was kept for further analyses. Consequently, SOPs from 9 RECs were obtained from the *HRWeb* platform, representing 8 African countries (Ethiopia, Lesotho, Malawi, Nigeria, South Africa, Togo, Uganda and Zambia) out of 54 (14,8 %).

Standard Operating Procedures from the Rwanda National Ethics Committee were obtained on the REC website, as advised by the Administrator of this REC. Hard copies or numeric files of SOPs were obtained from RECs in Burkina Faso, Cameroon, Egypt, Kenya, South Africa, and Tanzania, through personal contacts.

Finally, 16 SOPs from 14 African countries were collected from various sources as summarized in the flowchart (Fig. 1). Country, name of the REC, and name, year and source of the SOPs are detailed in Table 1. The year of publication of these SOPs was between 2007 and 2013 (*n* = 12) or not specified (*n* = 4).

**Table 1:** List of collected Standard Operating Procedures from African Research Ethics Committees

Country	REC name	SOP’s title	Year	source
Burkina Faso	Comité Institutionnel de Bioéthique du Centre National de Recherche et de Formation sur le Paludisme	POS du Comité Institutionnel de Bioéthique du Centre National de Recherche et de Formation sur le Paludisme	2013	hard copy personal comunication
Cameroon	Cameroon National Ethics Committee	SOPs for Research Ethics Committees in Cameroon	2012	numeric file personnal communication
Egypt	Magdi Yacoub Foundation	SOPs for Magdi Yacoub Foundation - Research Ethics Committee	2012	numeric file personnal communication
Ethiopia	Health Research Ethics Review Committee College of Health Sciences Mekelle University	Terms of Reference and Operating Procedures	n/a	numeric file from HRWeb
Kenya	Institutional Research and Ethics Committee of the Moi University College of Health Science and Moi Teaching and Referral Hospital	SOPs for Institutional Research and Ethics Committee	n/a	numeric file personnal communication
Lesotho	National Health Research Ethics Committee (NH-REC)	Standard Operating Procedures for NH-REC [Version 2 Draft]	2013	numeric file from HRWeb
Malawi	College of Medicine Research and Ethics Committee (COMREC)	General Guidelines on Health Research	2010	numeric file from HRWeb
Nigeria	Zeta-12 Independant Research Ethics Committee (ZIREC)	Zeta-12 Independant Research Ethics Committee (ZIREC) Mission Statement and Standard of Procedures	n/a	numeric file from HRWeb
Rwanda	Rwanda National Ethics Committee (RNEC)	Rwanda National Ethics Committee SOPs	2009	numeric file from website
South Africa	South African Medical Association Research Ethics Committee (SAMAREC)	SOPs and guidelines for the ethics evaluation of clinical trials in humans	2011	numeric file personnal communication
South Africa	Biomedical Research Ethics Committee (BREC) University of Kwazulu Natal	Biomedical Research Ethics Committee Terms of Reference & Standard Operating Procedures	2008	numeric file from HRWeb
South Africa	Human Research Ethics Committee	Human Research Ethics Committee Manual of SOPs	2009	numeric file from HRWeb
Tanzania	National Health Research Ethics Review Committee	SOPs for the National Health Research Ethics Review Committee	2007	book ISBN 9987 488-01-9 and website
Togo	Comité de Bioéthique pour la Recherche en Santé	Arrete N° 0153/2009/MS/CAB/DGS portant Charte du Comité de Bioéthique pour la Recherche en Santé	2009	numeric file from HRWeb
Uganda	Makerere University College of Health Sciences School of Medicine Research Ethics Committee (SOMREC)	SOMREC SOPs 1–14	2011	numeric file from HRWeb
Zambia	University of Zambia Biomedical Research Ethics Committee	University of Zambia Biomedical Research Ethics Committee SOPs	n/a	numeric file from HRWeb

*HRWeb* Health Research Web, *SOPs/POS* Standard Operating Procedures, *REC* Research Ethics Committee, *n/a* not available

### Qualitative analysis of collected standard operating procedures

**Fig. 2:**
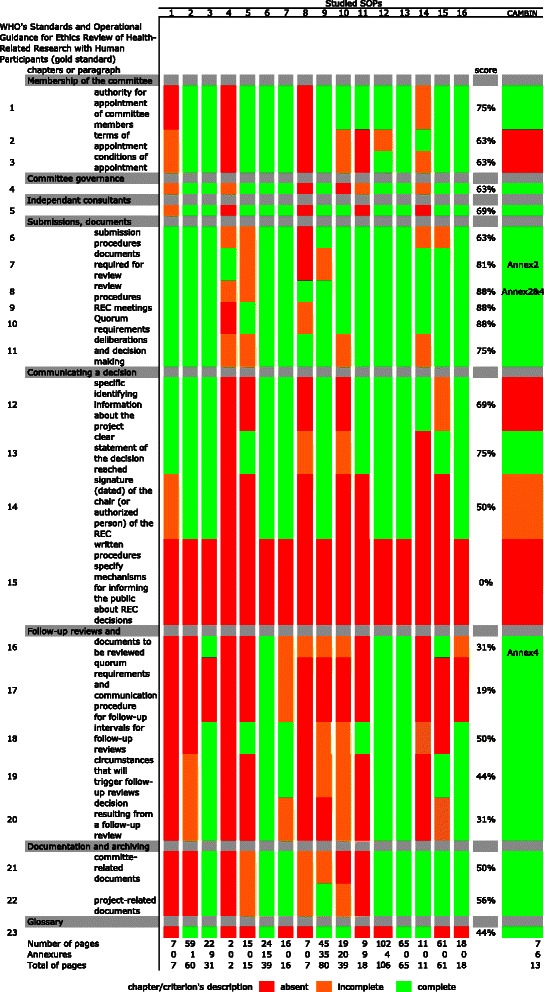
Qualitative analysis of collected Standard Operating Procedures from African Research Ethics Committees. The 16 African Standard Operating Procedures (SOPs) were qualitatively compared to the WHO guidelines (considered here as the gold standard). The score column represents the proportion of the 16 analyzed SOPs for which the description of a criterion was complete

The WHO's “Standards and Operational Guidance for Ethics Review of Health-Related Research with Human Participants” [3], used here as the gold standard, is composed of 8 chapters: members of the committee, committee governance, independent consultants, submission documents required, communicating a decision, follow-up reviews and monitoring, documentation and archiving, and glossary, and includes 23 criteria, as summarized in Fig. 2. The qualitative analysis of each of the 16 SOPs compared to the gold standard revealed a great diversity both in the quality and length of these SOPs. Standard Operating Procedures number 6 and 13 contained 22 out of 23 criteria with a complete description. Standard Operating Procedures number 4, 8 and 11, showed that 18, 15 and 12 criteria respectively were absent, whereas SOP number 10 recorded the highest rate of criteria with incomplete description (9 criteria out of 23). Concerning the length of the SOPs, it varied from 2 to 102 pages, excluding annexures (Fig. 2).

**Fig. 3:**
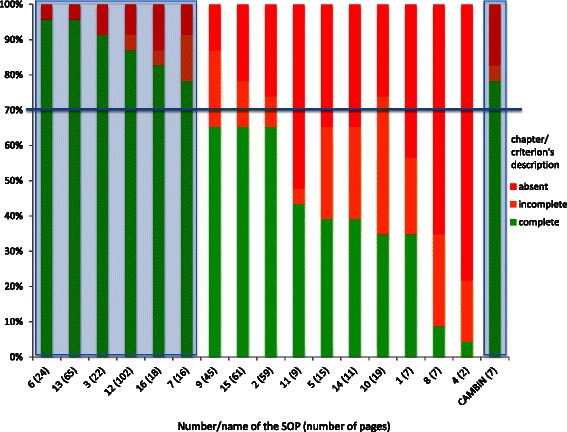
Decreasing qualitative classification of collected Standard Operating Procedures from African Research Ethics Committees. The 16 African Standard Operating Procedures (SOPs) were qualitatively compared to a gold standard (the WHO's guidelines) and then classified in a decreasing manner with regards to the proportion of fully described criteria. Of the 16 SOPs, SOPs number 6, 13, 3, 12, 16 and 7 had more than 70 % of criteria that were fully described (highlighted by a blue shadow on the left). The SOPs of the Ethics Review and Consultancy Committee of the Cameroon Bioethics Initiative (CAMBIN) were also above the threshold of 70 % (highlighted by a blue shadow on the right)

Standard Operating Procedures were then classified with regard to the proportion of complete description of criteria, as shown in Fig. 3. None of the SOPs contained a complete description of all the 23 criteria. The two SOPs that contained a complete description of 22 out of 23 criteria were of 24 and 65 pages long, respectively. Taking the threshold of 70 % of criteria that were fully described, we found 6 SOPs, of which the number of pages varied from 16 to 102 pages excluding annexures (Fig. 3).

**Fig. 4:**
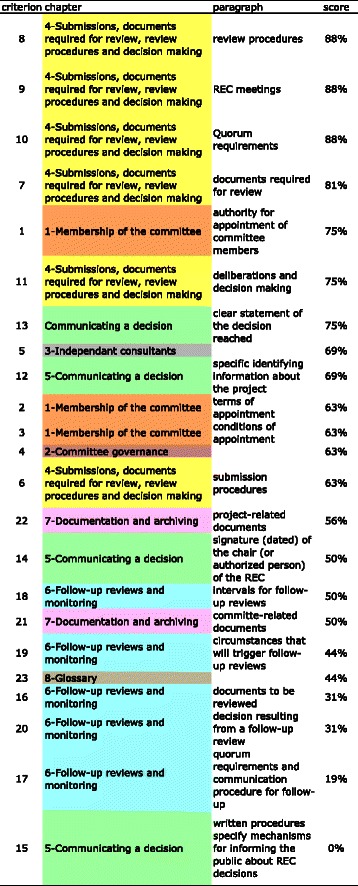
World Health Organization criteria sorted by score according to completeness of description in African Standard Operating Procedures. The 23 criteria of the WHO's guidelines were classified in a decreasing manner with regards to the proportion of Standard Operating Procedures (SOPs) that described completely each criterion. Each chapter of the WHO's guidelines was labeled with a specific color, for easy reading: 1-membership of the committee: orange, 2-Committee governance: maroon, 3-Independent consultant: grey, 4-Submissions, documents required for review, review procedures and decision making: yellow, 5-Communicating a decision: green, 6-Follow-up reviews and monitoring: blue, 7-Documentation and Archiving: mauve, and 8-Glossary: brown. Criteria 8, 9, 10 and 7 from chapter 4 ranked first, while score of criterion 15 (written procedures specify mechanism for informing the public about REC decision) reached 0 %

None of the criteria were completely described in all the SOPs. Information about chapter 4 on submissions, documents required for review, review procedures and decision-making was complete in most of the studied SOPs (Fig. 4). Particularly, criteria 8 (about review procedures) 9 (about REC meetings) and 10 (about quorum requirements) were completely described in 14 out of 16 SOPs (88 %). The authority for appointment of committee members was well described in 75 % of the SOPs, as well as a clear statement of the decision reached. The documentation and archiving chapter was completely described in about half of the SOPs (in 56 % of the SOPs for project related documents and in 50 % of the SOPs for committee related documents). The chapter 6 on the follow-up review and monitoring was fully described in only 19 to 50 % of the SOPs depending on the criteria. Finally, criterion 15 relating to mechanisms for informing the public about REC decisions was described in none of the 16 SOPs (Fig. 4).

### Analysis of the CAMBIN standard operating procedures

The CAMBIN SOPs [10] were then compared to the gold standard as we did for the 16 other SOPs. It appeared that criterion 14 (signature (dated) of the chair (or authorized person) of the REC) was partially described and criteria 2 (terms of appointment), 3 (conditions of appointment), 12 (specific identifying information about the project) and 15 (mechanisms for informing the public about REC decisions) were not mentioned (Fig. 2). However, the SOPs from the ERCC of the CAMBIN showed more than 78,3 % of criteria that were fully described (18 out of 23). Therefore, the CAMBIN SOPs were considered acceptable, like 6 other studied SOPs, as illustrated in Fig. 3.

## Discussion

Although all RECs/IRBs have the same primary mandate of providing ethical review of human research protocols so as to ensure that the dignity, rights, safety and wellbeing of research participants are protected, the organization of research ethics review varies from country to country and from one REC/IRB to another within the same country. In Africa, not all countries have a research review system, and a few do not even have as much as a single REC/IRB. At the time of the study and according to the *HRWeb* platform, 18 African countries (33 %) had no REC mapped. In Central Africa, for instance, this was the case for Chad and Equatorial Guinea. In addition, African RECs do not always have SOPs. In 2009, it was reported that 9 of 31 committees surveyed in Africa did not have SOPs and 7 of the committees that had SOPs had never revised their SOPs in a period of 3 years [11]. Similarly, in our study, we noticed that very few African RECs had SOPs (9 were obtained from the *HRWeb* platform and only 16 were collected in total), and that these SOPs, published between 2007 and 2013, had never been revised, or the revised version was not made available. Though this record may have improved very recently, it shows one of the major challenges of ethics committees in Africa.

Some African countries that have research review systems and RECs/IRBs have started to describe the regulation and organization of such systems and bodies recently [12, 13]. However, in the absence of legislation, the format describing how RECs/IRBs operate is very flexible: RECs/IRBs may choose to adopt the format and/or nomenclature of internal regulations, guidelines or SOPs, to describe their internal structure and operation. The heterogeneous format, content, and length of the SOPs that were analyzed in our study confirmed such flexibility (Table 1, Fig. 2).

Although these documents are legally non-binding instruments and may therefore not be strictly followed, they are important both at the external and internal levels. At the external level, the existence of such documents allows recognition of the REC/IRB not only by the national regulatory authority but also by global accreditation bodies such as the US federal wide assurance, and/or funding agencies. At the internal level, having internal regulations allows REC/IRB members to follow standardized procedures for receiving and reviewing research protocols and communicating with principal investigators in a systematic, non-ad hoc, non-trivial manner and this could help improve the overall functioning of the REC. Although REC/IRB records and decisions are for the most part confidential, it appears clearly useful that the documents describing the general functioning of RECs/IRBs be made publicly available, for the information and benefit of all actors involved in health research (investigators, institutions, potential participants, national health authorities, funding agencies, etc.). As an illustration, the National Institute for Medical Research in Tanzania published its SOPs as a book and on its website for the national health research ethics review committee in 2007, while few other RECs/IRBs have published their guidelines online via the *HRWeb* portal or their own website (Table 1). However, considering the small number of SOPs collected through this study, this trend is not yet very well spread in Africa.

In rhyme with the increasingly complex and challenging situation of research on humans, and the procedural canons being laid down by the most vocal experts of the new field, the SOPs of the various committees on the African continent have tended to be long and detailed, if not intricate and complex, and written in language requiring much time and concentration to read through, let alone understand. Actually, a number of initiatives have proposed templates that can be used by RECs/IRBs who want to develop SOPs, among which are the WHO's guidelines, used here as the gold standard. These templates, in most cases, provide guidance on the content of each procedure and the result therefore is a collection of SOPs, which are eventually voluminous. However, the volume of these templates is by no means a prescription of what the eventual SOPs should be. Moreover, ethics committee members in Africa generally are unremunerated volunteers who can afford only very limited time for committee meetings, let alone for reading through protocols and related materials and who, furthermore, are working within an oral rather than literate cultural background. For that reason it seemed to us crucial and critical to develop review procedures that are clear, simple, devoid of bureaucratic and technical details and addressing only situations and problems likely in our context. On the basis of this conviction, we embarked, as part of an EDCTP-funded project, in elaborating short, simple and clear SOPs for our committee, the ERCC of the CAMBIN, using as our guide the WHO's Standards and Operational Guidance for Ethics Review of Health-Related Research with Human Participants. It was our belief that our abbreviated and hopefully user-friendly SOPs, in relative terms, leave out little that is essential for rigorous and perfectly satisfactory protocol review and could serve as a good model and template for other committees in Africa or elsewhere in the developing world, that generally are facing the same situation and challenges with regards to credible, effective and timely protocol review.

In our study, among the 16 collected SOPs, the average length was of 30 pages (range: 2–102 pages). By comparison to the guidance of the WHO, only six of them were found acceptable/good with more than 70 % of the criteria from the gold standard that were fully described. Among those six, two of them were very long and detailed (65 and 102 pages), while the four remaining SOPs ranged from 16 to 24 pages. Since they were considered qualitatively as equivalent (Fig. 3), shorter SOPs would be preferable compared to longer ones, particularly in the African context described above.

Although we used the WHO's guidelines to elaborate the CAMBIN SOPs, a few criteria were still missing as revealed by this systematic analysis. For example, our SOPs scored zero in relation to criterion number 2. However, this is not because CAMBIN ERCC doesn’t have a practice for the terms of appointment of its members but because these terms had not been written in the SOP. We realized that they would then need to be revised/ improved/updated in the near future in order to include missing criteria as evidenced by comparison to the gold standard or to address unforeseen/unforeseeable developments in the domain. This revision process is currently being carried out. Nevertheless, the CAMBIN SOPs were considered as acceptable/good with 78.3 % (18/23) of the criteria from the gold standard that were fully described. In addition, these SOPs were the shortest SOPs (7 pages) when compared to the 6 other acceptable SOPs that had 13, 18, 22, 24, 65 or even 102 pages long. When we looked at the shortest available SOPs from our study, we found that 5 SOPs had less than 12 pages (between 2 and 11 pages). However, none of these was considered as acceptable in terms of quality. Indeed, these SOPs had only between 4,3 % and 43,5 % of the required criteria that were fully described (Fig. 3). Because they do not always meet the core elements of the gold standard, short SOPs may not be promoted at all costs. Future revisions/updates of our SOPs will probably tend to lengthen rather than shorten them. But the advantage here would be a growth stimulated by contextual imperatives and relevance with implications for originality and ownership. That way the SOPs are likely to have a slow organic growth that enhances the comprehension and familiarity of the users.

Publicly available on the CAMBIN ERCC website [10], the current content of our SOPs is as follows: i) purpose, ii) scope, iii) governance: this procedure states and describes the roles of the principal officers of the ERCC. It also addresses the role of independent consultants who could be invited by the ERCC if the need arises, iv) procedures: this describes the application process, expedited review, the review process, conflict of interest, committee decisions, and documentation, v) appendix: this provides basic documents that could be of relevance to committee members and investigators who plan to submit or have submitted an application for ethics clearance to the CAMBIN ERCC; these include: conflict of interest statement, checklist of the documents to be submitted by an applicant, site visit form for committee members, checklist for protocol review, and sample report form for the principal investigator, vi) revision history: it is understood that new ethical issues in health research will keep emerging thereby necessitating the need for revisions of these SOPs to maintain their currency and relevance. This procedure therefore states the version of the SOPs, the year it was revised and the description of changes made in the preceding SOPs, and vii) glossary: this section of the SOPs lists in alphabetical order and defines some technical terms used in the entire document.

Interestingly, the CAMBIN ERCC SOPs are neither longer nor more complex than the Declaration of Helsinki. They are written in a language any conscientious twelfth-grader (12 years of formal schooling) could easily comprehend. We strove for brevity, clarity and simplicity, in order to make them more user-appropriate in our specific context, marked by resource poverty and sundry cultural impediments to habitual reading and writing. Actually, our SOPs were elaborated closely following the annex 3 of the WHO’s guidelines, which is only 9 pages long. We believe that our abridged, user-friendly SOPs leave out little that is essential for a smooth functioning of an ethics review committee or for rigorous and satisfactory protocol review. The CAMBIN ERCC SOPs appear to us a good compromise in terms of quality/brevity, complexity/comprehension and an acceptable model for other RECs that are similarly situated. The results of this qualitative analysis allow us, therefore, to propose our SOPs as a model and template for similarly situated ethics committees, especially those on the African continent.

## Conclusions

While RECs in Africa have generally striven to fulfill the requirement of multidisciplinarity in their membership composition, lack of resources does not generally permit training in ethics review, not to mention ensuring that adequate time and attention would be devoted to review tasks. Therefore, it appeared crucial to develop SOPs that are adapted to that context.

Although SOPs of 7 pages may appear too small when compared to most extant SOPs, we believe and argue that these SOPs address the essential procedures of a REC and because of their brevity and clarity, they should be relatively easier for applicants and committee members to comprehend and follow. We recommend that RECs in Africa and elsewhere in the developing world cut down drastically on the number pages (not necessarily content) of their SOPs, so as to enable committee members, investigators and other persons interested in the activity/service of a REC/IRB to easily read and comprehend the procedures. While long and detailed SOPs, may have clear advantages for legally-minded people and those who want to exclude the possibility of incurring any blame in their actions, we are convinced that, because of their brevity, simplicity, clarity and user-friendliness, the ERCC SOPs recommend themselves as a model template to, at least, committees similarly situated and/or circumstanced as the ERCC of the Cameroon Bioethics Initiative is. In fact, brevity, clarity, simplicity and user-friendliness are recognized values. Whatever is brief and clear is better than what is not and saves time. What is simple and user-friendly is better than what is not even though the two have the same aims because it saves both time and mental energy. And if this be true in general, it is even truer of the context and its peculiar constraints that we are addressing. We believe that small is also beautiful and that the advantages of simple, succinct SOPs within the context of our present situation, by far outweigh any disadvantages.

### Ethics approval and consent to participate

Not applicable.

### Consent for publication

Not applicable.

### Availability of data and materials

Given the fact that the SOPs were numbered to preserve anonymity for further analyses and presentation of results, we have elected not to make the raw data publicly available. However, each IRB whose SOPs were analyzed can get its own result on request to the authors.

## Abbreviations

CAMBIN: Cameroon Bioethics Initiative
COHRED: Council On Health Research for Development
ERCC: Ethics Review and Consultancy Committee
HRWeb: Health Research Web
IRB: Institutional Review Board
REC: Research Ethics Committee
SOP: Standard Operating Procedures
WHO: World Health Organization
